# Distribuição dos Centros de Especialidades Odontológicas nas regiões de saúde do Brasil, 2023

**DOI:** 10.1590/0102-311XPT165425

**Published:** 2026-07-06

**Authors:** Iêda Lenzi Durão, Marla Presa Raulino Schilling, Margareth Crisóstomo Portela

**Affiliations:** 1 Escola Nacional de Saúde Pública Sergio Arouca, Fundação Oswaldo Cruz, Rio de Janeiro, Brasil.

**Keywords:** Atenção Secundária, Regionalização da Saúde, Serviços de Saúde Bucal, Secondary Care, Regional Health Planning, Dental Health Services, Atención Secundaria, Regionalización, Servicios de Salud Dental

## Abstract

Este estudo propõe-se a analisar a distribuição dos Centros de Especialidades Odontológicas (CEO) nas regiões de saúde brasileiras e identificar fatores contextuais associados a essa distribuição. Trata-se de um estudo ecológico transversal baseado em dados secundários de 1.214 CEO ativos em dezembro de 2023, extraídos do Cadastro Nacional de Estabelecimentos de Saúde. Utilizou-se o modelo *hurdle* binomial negativo para identificar os fatores associados à distribuição desses serviços, considerando características demográficas e socioeconômicas, bem como a disponibilidade de equipes de saúde bucal na atenção primária. Os resultados mostraram que 82,44% das regiões de saúde do Brasil têm pelo menos um CEO. A Região Nordeste destacou-se pela presença de CEO em 93,23% das regiões de saúde, superando as demais regiões. O índice de vulnerabilidade social mostrou-se associado à presença e à contagem de CEO, com efeitos paradoxais. As regiões com maior vulnerabilidade apresentaram menores chances de ter CEO (OR = 0,905; IC95%: 0,871-0,940), mas quando apresentavam pelo menos um CEO, observou-se aumento na quantidade desses serviços (RT = 1,023; IC95%: 1,013-1,033). Embora a implementação dos CEO ocorra predominantemente em regiões menos vulneráveis, estão sendo desenvolvidas estratégias compensatórias para que esses serviços estejam disponíveis à população de regiões mais vulneráveis (hipótese da equidade inversa). Contudo, persistem desafios significativos relacionados à dimensão territorial brasileira, aos vazios assistenciais e à necessidade de otimizar o uso dos CEO. Ainda que seja necessário pensar a atenção secundária em saúde bucal em um contexto regionalizado, a desigual distribuição de regiões de saúde entre os estados e regiões geográficas, a variação na área territorial e as dificuldades de mobilidade nessas regiões podem afetar o acesso aos CEO.

## Abstract (en)

This study aims to analyze the distribution of Dental Specialty Centers (CEOs, acronym in Portuguese) across Brazilian health regions and to identify contextual factors associated with this distribution. This ecological cross-sectional study used secondary data from 1,214 active CEOs in December 2023, extracted from the Brazilian National Registry of Health Establishments. The hurdle negative binomial model was applied to identify factors associated with the distribution of these services, considering demographic and socioeconomic characteristics, as well as the availability of oral health teams in primary care. The results showed that 82.44% of Brazil’s health regions have at least one CEO. The Northeast stood out, with 93.23% of its health regions housing CEOs, exceeding proportions observed in other regions. The social vulnerability index was associated with both the presence and the number of CEOs, showing paradoxical effects. Regions with higher vulnerability were less likely to have a CEO (OR = 0.905; 95%CI: 0.871-0.940), but among regions with at least one CEO, higher vulnerability was associated with an increased number of these services (RR = 1.023; 95%CI: 1.013-1.033). Although CEOs are predominantly implemented in less vulnerable regions, compensatory strategies appear to be emerging to ensure service availability in more vulnerable areas, consistent with the inverse equity hypothesis. However, significant challenges remain regarding Brazil’s territorial dimension, gaps in service coverage, and the need to optimize the use of CEOs. Although secondary oral healthcare must be considered within a regionalized context, the unequal distribution of health regions across states and geographic regions, variation in territorial area, and mobility constraints may affect access to CEOs.

## Abstract (es)

Este estudio se propone analizar la distribución de los Centros de Especialidades Odontológicas (CEO) en las regiones sanitarias brasileñas e identificar los factores contextuales asociados a dicha distribución. Se trata de un estudio ecológico transversal basado en datos secundarios de 1.214 CEO activos en diciembre de 2023, extraídos del Catastro Nacional de Establecimientos de Salud. Se utilizó el modelo *hurdle* binomial negativo para identificar los factores asociados a la distribución de estos servicios, teniendo en cuenta las características demográficas y socioeconómicas, así como la disponibilidad de equipos de salud bucal en la atención primaria. Los resultados mostraron que el 82,44% de las regiones sanitarias de Brasil cuentan con al menos un CEO. La Región Nordeste destacó por la presencia de CEO en el 93,23% de las regiones sanitarias, superando al resto de regiones. El índice de vulnerabilidad social se mostró asociado a la presencia y el recuento de CEO, con efectos paradójicos. Las regiones con mayor vulnerabilidad presentaron menores probabilidades de contar con un CEO (OR = 0,905; IC95%: 0,871-0,940), pero cuando contaban con al menos un CEO, se observó un aumento en la cantidad de estos servicios (RT = 1,023; IC95%: 1,013-1,033). Aunque la implementación de los CEO se produce predominantemente en regiones menos vulnerables, se están desarrollando estrategias compensatorias para que estos servicios estén disponibles para la población de las regiones más vulnerables (hipótesis de la equidad inversa). Sin embargo, persisten retos significativos relacionados con la dimensión territorial brasileña, las carencias asistenciales y la necesidad de optimizar el uso de los CEO. Aunque es necesario pensar en la atención secundaria en salud bucodental en un contexto regionalizado, la distribución desigual de las regiones sanitarias entre los estados y las regiones geográficas, la variación en la superficie territorial y las dificultades de movilidad en estas regiones pueden afectar al acceso a los CEO.

## Introdução

A implementação da Política Nacional de Saúde Bucal (PNSB), em 2004, representou um marco transformador no acesso aos serviços odontológicos pelo Sistema Único de Saúde (SUS), por meio da ampliação das equipes de saúde bucal (eSB) junto à Estratégia Saúde da Família (ESF), da implantação dos Centros de Especialidades Odontológicas (CEO) e dos Laboratórios Regionais de Prótese Dentária (LRPD). Esta política rompeu com o modelo excludente de atenção odontológica no Brasil, que atendia, prioritariamente, a crianças de até 12 anos e tem conseguido alcançar uma população historicamente desfavorecida e excluída da atenção em saúde bucal ^1^.

Nesse contexto de expansão da rede de atenção em saúde bucal no SUS, é fundamental considerar a estrutura, em termos de recursos físicos, humanos e financeiros, como elemento fundamental para a provisão e a qualidade do cuidado, pois constitui a base física e organizacional sobre a qual os processos assistenciais são desenvolvidos ^2^. A disponibilidade de uma estrutura adequada não garante, mas favorece a realização de processos de cuidado mais adequados e melhores resultados ^2^ ^,^ ^3^ ^,^ ^4^, mostrando-se associada à efetividade dos serviços e à satisfação dos usuários ^5^.

A partir de 2006, com a instituição do financiamento para a implantação dos CEO ^6^, houve um aumento destes serviços no Brasil. No ano seguinte, foram credenciados 498 CEO e, em 2023, havia 1.214 CEO ^7^. Da mesma forma, houve o crescimento de eSB. Antes da criação da PNSB, em 2003, havia 6.393 eSB. Em 2023, o número de eSB cadastradas passou para 39.5437. A expansão de eSB foi mais acentuada em municípios de menor porte populacional e menor produto interno bruto (PIB) *per capita* ^8^, enquanto os CEO foram implantados em municípios de maior porte populacional e de maior desenvolvimento econômico ^9^.

A consolidação jurídica desta política ocorreu em 2023, quando a *Lei nº 14.572/2023* foi sancionada, incorporando formalmente a PNSB à Lei Orgânica da Saúde (*Lei nº 8.080/1990*) ^10^. Esta medida legislativa fortaleceu a garantia do acesso universal, equitativo e contínuo aos serviços odontológicos, alinhando-se diretamente ao objetivo de atingir a cobertura universal de saúde até 2030 estabelecido pela Organização das Nações Unidas (ONU) em seus *Objetivos de Desenvolvimento Sustentável* (ODS) ^11^. Com esta mudança normativa, a saúde bucal transcendeu o *status* de programa governamental para se tornar um direito assegurado por lei a todos os brasileiros. Esse avanço confere o protagonismo necessário para o desenvolvimento de ações efetivas voltadas à integralidade do cuidado, bem como à ampliação da rede assistencial ^10^.

Os CEO integram a Rede de Atenção à Saúde Bucal (RASB) ^12^ e podem ser de três tipos: CEO I (3 cadeiras), CEO II (4 a 6 cadeiras) e CEO III (mais de 7 cadeiras) ^13^. Estas unidades desempenham papel fundamental na garantia da integralidade do cuidado ^1^ ^,^ ^14^ e configuram-se como elementos estratégicos na estruturação da atenção secundária em saúde bucal ^9^ ^,^ ^15^, oferecendo, obrigatoriamente, cinco especialidades essenciais: cirurgia oral, estomatologia, periodontia, endodontia e atendimento a pacientes com necessidades especiais (PNE). A decisão de credenciamento desses serviços depende do interesse do gestor municipal ou estadual e deve ter como um dos critérios uma ampla cobertura por eSB. Além disso, deve demonstrar coerência com o Plano Diretor de Regionalização e identificar a área de abrangência (município, região ou microrregião de saúde), bem como a população a ser atendida ^13^.

Apesar dos avanços significativos com a implantação dos CEO, persistem desafios que comprometem o desempenho potencial destes serviços e a resposta às necessidades da população brasileira ^16^ ^,^ ^17^. A literatura científica evidencia um paradoxo preocupante. Os CEO, embora insuficientes para atender a toda a demanda existente, apresentam, simultaneamente, uma subutilização de sua capacidade instalada ^18^ ^,^ ^19^. Este fenômeno contraditório tem raízes multifatoriais, incluindo deficiências na gestão dos serviços ^18^ e falhas na articulação entre a atenção primária e a secundária em saúde bucal ^20^. Observa-se, ainda, que grande parte dos estudos sobre o desempenho e o cumprimento de metas de produção dos CEO ^9^ ^,^ ^18^ ^,^ ^21^ ^,^ ^22^ ^,^ ^23^ ^,^ ^24^ evidencia dificuldades quanto à capacidade dos municípios de atender às necessidades acumuladas de saúde bucal da população. Análises sobre a distribuição de CEO identificaram desigualdades regionais ^20^ e a associação entre maior disponibilidade de serviços especializados e piores indicadores socioeconômicos municipais ^25^.

Considerando o panorama apresentado, lacunas de conhecimento sobre a organização regional dos CEO se colocam diante do crescimento expressivo da atenção secundária em saúde bucal no Brasil na última década - cerca de 23% entre 2014 (931 CEO) e 2023 (1.214 CEO) - somado ao fato de estudos pregressos focarem municípios com CEO implantados, como unidade de análise ^20^ ^,^ ^25^. A abordagem regional no planejamento da saúde bucal é importante para a consolidação da PNSB ^25^ ^,^ ^26^ ^,^ ^27^, embora existam obstáculos decorrentes das desigualdades territoriais na distribuição de recursos físicos, humanos e financeiros, que comprometem a regionalização e o acesso equitativo ^27^ ^,^ ^28^.

Este trabalho parte do entendimento de que as regiões de saúde são espaços geográficos que integram a organização, o planejamento e a execução de ações de saúde ^29^ e de que a regionalização é central para a oferta eficiente de recursos de saúde mais complexos ^12^ ^,^ ^29^. Assim, a atenção especializada, como parte da estrutura da RASB ^12^, deveria estar presente em todas as regiões de saúde, e o credenciamento dos CEO deveria demonstrar coerência com o Plano Diretor de Regionalização ^13^. Pretende-se abordar a inserção da atenção secundária em saúde bucal no contexto da regionalização, fator importante para a implantação dos CEO ^28^ e para o aumento do acesso da população aos serviços especializados ^30^.

Este estudo propõe-se a analisar a distribuição dos CEO, cadeiras odontológicas e cirurgiões-dentistas nas regiões de saúde brasileiras e a identificar fatores contextuais associados a essa distribuição, considerando características socioeconômicas e demográficas, bem como a disponibilidade de eSB.

## Métodos

### Desenho do estudo

O estudo caracteriza-se como ecológico, de caráter descritivo e analítico, com abordagem transversal e abrangência nacional, baseado em dados secundários públicos de acesso irrestrito. A extração e o gerenciamento dos dados ocorreram entre junho e novembro de 2024.

### Critério de inclusão

Foram incluídos no estudo os 1.214 CEO ativos em dezembro de 2023, segundo dados do Cadastro Nacional de Estabelecimentos de Saúde (CNES) ^31^.

### Fontes e coleta de dados

Para a obtenção das informações relativas à disponibilidade de CEO, cadeiras odontológicas e cirurgiões-dentistas de atenção secundária em saúde bucal foram utilizados tabelas e relatórios do Departamento de Informação e Informática do SUS (DATASUS) referentes ao CNES ^31^, em dezembro de 2023. Por meio do cruzamento de tabelas e relatórios, foi possível extrair informações sobre a estrutura dos CEO, que foram trabalhadas no Colab Notebook (https://colab.research.google.com/), disponibilizado pelo Google, em Python.

Informações demográficas e socioeconômicas foram obtidas do Instituto Brasileiro de Geografia e Estatística (IBGE) ^32^ e do Instituto de Pesquisa Econômica Aplicada (IPEA) ^33^. As informações referentes a eSB foram coletadas por meio dos relatórios da atenção primária em saúde (APS) ^34^.

Todo o processo envolveu múltiplas operações de filtragem e agrupamento para consolidar um banco de dados com informações demográficas, socioeconômicas e de atenção primária e secundária em saúde bucal, por município, e, posteriormente, realizar o agrupamento por regiões de saúde. Informações detalhadas sobre a metodologia e a extração de dados estão disponíveis em: https://data.mendeley.com/datasets/fb22pnxkyr/2.

### Variáveis

O estudo analisou a distribuição de três componentes da estrutura da atenção secundária em saúde bucal por região de saúde: (1) número de CEO, (2) número de cadeiras odontológicas em CEO e (3) número de cirurgiões-dentistas em CEO. O número de CEO foi definido como a variável de desfecho para a análise multivariada. As variáveis explicativas incluíram: densidade demográfica (2022), Índice de Vulnerabilidade Social ‒ IVS (2010), densidade de eSB (2023), macrorregiões do país, Índice de Desenvolvimento Humano Municipal ‒ IDHM (2010), cobertura de eSB (2023) e cobertura de água fluoretada (2017). A inclusão das macrorregiões fundamentou-se na hipótese de desigualdades regionais na distribuição de CEO, com maior oferta esperada em regiões de maior densidade demográfica, melhores indicadores socioeconômicos e serviços de atenção primária em saúde bucal mais consolidados.

Para o cálculo do IVS foi realizado o agrupamento ponderado pela população, com vistas a refletir a realidade social e econômica da região de saúde como um todo. A ponderação permite que os indicadores representem de forma mais adequada a situação vivida pela maioria da população, corrigindo distorções que ocorreriam caso todos os municípios ou áreas tivessem o mesmo peso no cálculo, independentemente de seu tamanho populacional ^35^.

Todas as variáveis foram extraídas por município e, posteriormente, agrupadas pelas 450 regiões de saúde do país. Com vistas à apresentação de um panorama da distribuição de CEO, cadeiras odontológicas e cirurgiões-dentistas da atenção secundária em saúde bucal nas Unidades Federativas (UF) e nas macrorregiões do país, também foram realizados agrupamentos nesses níveis de análise - população, extensão territorial, densidade demográfica, número de regiões de saúde e disponibilidade de CEO, cadeiras odontológicas e cirurgiões-dentistas.

### Análises

Realizaram-se análises descritivas para caracterizar a distribuição de CEO, cadeiras odontológicas e cirurgiões-dentistas da atenção secundária em saúde bucal nas macrorregiões e nas UF. Foram calculadas taxas de disponibilidade por 100 mil habitantes para CEO e cadeiras odontológicas e cirurgiões-dentistas em CEO. Também foram apresentados aspectos demográficos e territoriais, incluindo a presença de CEO e a distribuição das regiões de saúde. Em seguida, foi realizada a análise da distribuição das regiões de saúde conforme as taxas de disponibilidade desses recursos.

Considerando a natureza e a distribuição do desfecho - total de CEO na região de saúde -, aplicou-se um modelo *hurdle* binomial negativo, apropriado para lidar com excesso de zeros e com variância superior à média. O modelo utiliza um processo de modelagem em dois estágios: (1) parte binária, que estima as chances de uma região ter ao menos um CEO (*logit*); e (2) parte de contagem, que estima o número de CEO, condicionado à existência de pelo menos um CEO (contagem positiva), a partir de um modelo binomial negativo truncado em zero ^36^ ^,^ ^37^. Este tipo de análise apresenta como principal vantagem uma grande flexibilidade, pois permite que o processo que determina a ausência do serviço (zero) seja diferente do que determina a magnitude das contagens positivas. Tem sido utilizado para estudar o uso dos serviços de saúde ^36^ ^,^ ^37^ ^,^ ^38^ ^,^ ^39^ e para avaliar políticas públicas ^36^.

Para o primeiro processo (disponibilidade *vs.* indisponibilidade de CEO na região de saúde), foram incluídos, no modelo final, os seguintes preditores: o IVS, as macrorregiões do Brasil, tendo como referência a Região Sudeste, e a densidade de eSB (eSB/100km^2^). Para o segundo processo (contagem de CEO, dada a sua disponibilidade na região de saúde), foram incluídos o IVS e a densidade demográfica.

As variáveis IDHM (2010), cobertura de eSB (2023) e cobertura de água fluoretada (2017) foram inicialmente avaliadas, porém excluídas do modelo final. Todas as variáveis testadas são amplamente descritas na literatura como fatores associados à implementação de serviços de saúde. Cada variável foi incluída individualmente, e apenas as estatisticamente significativas permaneceram no modelo final.

Empregou-se como *offset* a população da região de saúde, a fim de ajustar as contagens brutas às diferenças no tamanho populacional. Para uma interpretação mais compreensível dos resultados na análise multivariada, a variável IVS foi multiplicada por 100, com uma unidade da variável modificada expressando 1 centésimo da original; e a variável densidade demográfica foi dividida por 100, com uma unidade da variável modificada expressando uma centena de indivíduos por km^2^. O nível de significância estatística adotado foi de 5%. As análises foram realizadas no software R (http://www.r-project.org) e utilizaram-se os pacotes *pscl*, *DHARMa*, *performance* e *glmmTMB*.

### Aspectos éticos

A pesquisa utilizou informações de domínio público, disponibilizadas de forma irrestrita pelo DATASUS, pelo IBGE e pelo IPEA. Houve dispensa de apreciação por Comitê de Ética em Pesquisa da Fundação Oswaldo Cruz (parecer nº 16/2024), conforme a *Resolução nº 510/2016* do Conselho Nacional de Saúde (CNS). A pesquisa atendeu às *Resoluções nº 196/1996*, *nº 466/2012* e *nº 510/2016* do CNS e foi conduzida de forma ética e legal ^40^ ^,^ ^41^ ^,^ ^42^.

## Resultados

Ao analisar os dados sobre a distribuição de CEO, cadeiras odontológicas e cirurgiões-dentistas de atenção secundária em saúde bucal no SUS no país (Tabela 1), verifica-se heterogeneidade entre as diferentes regiões e estados brasileiros. Em dezembro de 2023, o Brasil tinha 1.214 CEO habilitados no CNES, distribuídos em 371 (82,44%) das 450 regiões de saúde. Observa-se que apenas oito estados (Mato Grosso do Sul, Alagoas, Ceará, Paraíba, Pernambuco, Rio Grande do Norte, Espírito Santo e Rio de Janeiro) e o Distrito Federal tinham 100% de suas regiões de saúde cobertas por CEO.

A Região Nordeste apresenta elevada presença da atenção secundária em saúde bucal em suas regiões de saúde (93,23%), com destaque para a Paraíba, que tem os melhores indicadores: 2,72 CEO/100 mil habitantes, 7,98 cadeiras odontológicas em CEO/100 mil habitantes e 19,20 cirurgiões-dentistas em CEO/100 mil habitantes. Em contrapartida, a Região Norte apresenta o pior panorama, com menos de 70% de suas regiões de saúde com CEO.

**Tabela 1:** Distribuição de recursos da atenção secundária em saúde bucal no Sistema Único de Saúde (SUS) por regiões e Unidades Federativas (UF) do Brasil, 2023.

Região/UF	População [n (%)]	Área territorial [km^2^ (%)]	Densidade demográfica (habitantes/km^2^)	Quantidade de regiões de saúde	Regiões de saúde com CEO [n (%)]	CEO (por 100 mil habitantes)	Cadeiras odontológicas em CEO (por 100 mil habitantes)	Cirurgião dentista em CEO (por 100 mil habitantes)
Norte	17.354.884 (8,55)	3.850.373 (45,33)	4,51	45	31 (68,89)	0,44	1,88	5,38
Acre	830.018 (0,41)	164.162 (1,93)	5,06	3	1 (33,33)	0,24	1,57	5,18
Amazonas	3.941.613 (1,94)	1.559.228 (18,36)	2,53	9	5 (55,56)	0,33	1,70	3,60
Amapá	733.759 (0,36)	142.462 (1,68)	5,15	3	2 (66,67)	0,55	3,41	18,81
Pará	8.120.131 (4,00)	1.245.800 (14,67)	6,52	13	10 (76,92)	0,49	1,80	5,07
Rondônia	1.581.196 (0,78)	237.729 (2,80)	6,65	7	5 (71,43)	0,51	2,21	3,48
Roraima	636.707 (0,31)	223.635 (2,63)	2,85	2	1 (50,00)	0,31	0,94	5,81
Tocantins	1.511.460 (0,74)	277.357 (3,27)	5,45	8	7 (87,50)	0,46	2,32	7,01
Nordeste	54.658.515 (26,91)	1.551.289 (18,26)	35,23	133	124 (93,23)	0,90	3,62	7,74
Alagoas	3.127.683 (1,54)	27.778 (0,33)	112,60	10	10 (100,00)	0,80	2,59	7,03
Bahia	14.141.626 (6,96)	564.552 (6,65)	25,05	28	26 (92,86)	0,59	2,38	5,32
Ceará	8.794.957 (4,33)	148.804 (1,75)	59,10	22	22 (100,00)	0,93	5,09	7,57
Maranhão	6.776.699 (3,34)	329.544 (3,88)	20,56	19	14 (73,68)	0,47	1,77	3,95
Paraíba	3.974.687 (1,96)	56.359 (0,66)	70,52	16	16 (100,00)	2,72	7,98	19,20
Pernambuco	9.058.931 (4,46)	97.978 (1,15)	92,46	12	12 (100,00)	0,86	3,52	9,21
Piauí	3.271.199 (1,61)	251.645 (2,96)	13,00	11	10 (90,90)	1,04	4,89	9,35
Rio Grande do Norte	3.302.729 (1,63)	52.728 (0,62)	62,64	8	8 (100,00)	1,12	4,24	9,33
Sergipe	2.210.004 (1,09)	21.901 (0,26)	100,91	7	6 (85,71)	0,59	2,53	6,83
Centro-oeste	16.289.538 (8,02)	1.606.131 (18,91)	10,14	39	28 (71,79)	0,56	3,72	8,84
Distrito Federal	2.817.381 (1,39)	5.760 (0,07)	489,13	1	1 (100,00)	0,50	5,86	12,10
Goiás	7.056.495 (3,47)	340.128 (4,00)	20,75	18	16 (88,89)	0,61	3,06	7,99
Mato Grosso do Sul	2.757.013 (1,36)	357.103 (4,20)	7,72	4	4 (100,00)	0,65	3,84	12,01
Mato Grosso	3.658.649 (1,80)	903.140 (10,63)	4,05	16	7 (43,75)	0,44	3,25	5,58
Sudeste	84.840.113 (41,78)	923.726 (10,87)	91,85	165	138 (83,64)	0,49	2,75	7,07
Espírito Santo	3.833.712 (1,89)	46.038 (0,54)	83,27	4	4 (100,00)	0,29	1,25	3,81
Minas Gerais	20.539.989 (10,11)	586.099 (6,90)	35,05	89	67 (75,28)	0,54	3,75	8,34
Rio de Janeiro	16.055.174 (7,91)	43.705 (0,51)	367,35	9	9 (100,00)	0,54	2,60	9,06
São Paulo	44.411.238 (21,87)	247.884 (2,92)	179,16	63	58 (92,06)	0,47	2,48	6,11
Sul	29.937.706 (14,74)	563.072 (6,63)	53,17	68	50 (73,53)	0,46	3,48	6,12
Paraná	11.444.380 (5,64)	199.108 (2,34)	57,48	22	18 (81,82)	0,45	4,95	6,18
Rio Grande do Sul	10.882.965 (5,36)	268.382 (3,16)	40,55	30	17 (56,67)	0,34	2,14	4,75
Santa Catarina	7.610.361 (3,75)	95.582 (1,13)	79,62	16	15 (93,75)	0,66	3,18	7,99
Brasil	203.080.756 (100,00)	8.494.591 (100,00)	23,91	450	371 (82,44)	0,60	3,09	7,11

CEO: Centros de Especialidades Odontológicas.

Na Região Centro-oeste, o Estado do Mato Grosso do Sul apresenta 100% das suas regiões de saúde (n = 4) cobertas e bons indicadores de disponibilidade de CEO, cadeiras odontológicas e cirurgiões-dentistas (0,65 CEO/100 mil habitantes, 3,84 cadeiras odontológicas em CEO/100 mil habitantes e 12,01 cirurgiões-dentistas em CEO/100 mil habitantes), distribuídos em uma grande área territorial (357.103km^2^).

Na Região Sul, o Estado do Rio Grande do Sul apresenta um dos menores percentuais de CEO: apenas 56,67% de suas regiões de saúde têm disponibilidade de CEO, bem abaixo da média nacional de 82,44% e da média da Região Sul (73,53%). A Região Sudeste, apesar de concentrar a maior parte da população brasileira (41,78%), apresenta indicadores de disponibilidade de CEO inferiores aos da Região Nordeste.

A Tabela 2 apresenta a distribuição das regiões de saúde segundo as taxas de disponibilidade, por 100 mil habitantes, de CEO, cadeiras odontológicas e cirurgiões-dentistas. Os resultados apontaram que 79 (17,6%) regiões de saúde não apresentam CEO e que a maioria (56,2%) dispõe de até um CEO por 100 mil habitantes. Com relação à disponibilidade de cadeiras odontológicas, observa-se que mais da metade (58,2%) das regiões de saúde apresenta entre um e cinco cadeiras odontológicas por 100 mil habitantes; aproximadamente 30% das regiões de saúde têm de cinco a 10 cirurgiões-dentistas atuantes em CEO.

**Tabela 2:** Distribuição das regiões de saúde (N = 450) segundo taxas de disponibilidade de recursos da atenção secundária em saúde bucal por 100 mil habitantes. Brasil, 2023.

Recurso da atenção secundária em saúde bucal	Taxa de disponibilidade por 100 mil habitantes													
	0		0┤1		1┤5		5┤10		10┤15		15┤20		> 20	
	n	%	n	%	n	%	n	%	n	%	n	%	n	%
CEO	79	17,6	253	56,2	113	25,1	5	1,1	-	-	-	-	-	-
Cadeiras odontológicas em CEO	79	17,6	16	3,6	262	58,2	70	15,6	13	2,9	7	1,6	3	0,7
Cirurgiões-dentistas em CEO	79	17,6	1	0,2	99	22,0	141	31,3	70	15,6	31	6,9	29	6,4

CEO: Centros de Especialidades Odontológicas.

**Figura 1:**
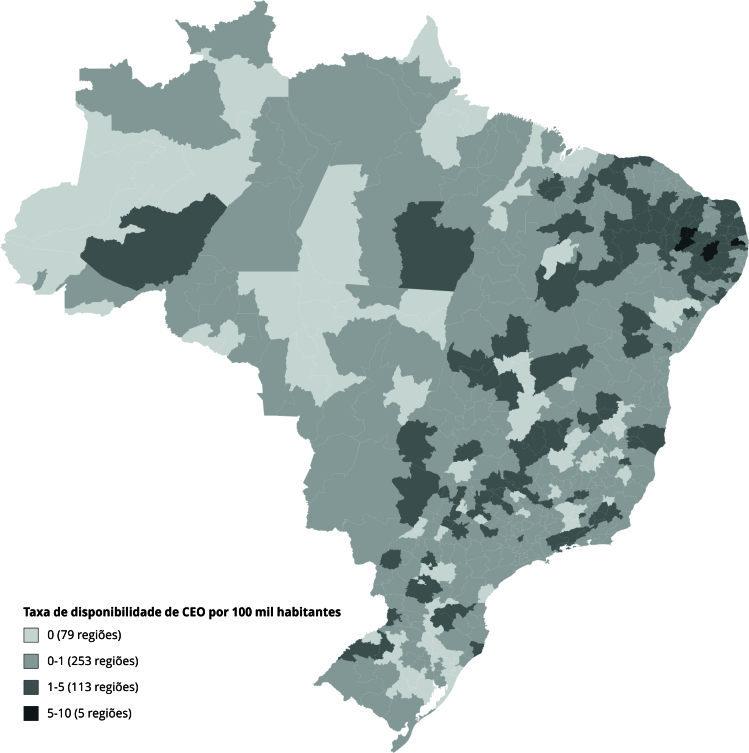
Distribuição de Centros de Especialidades Odontológicas (CEO) segundo região de saúde. Brasil, 2023.

A Figura 1 ilustra as taxas de disponibilidade de CEO nas regiões de saúde do país. Com relação ao número absoluto de CEO, cadeiras odontológicas e cirurgiões-dentistas da atenção secundária em saúde bucal nas regiões de saúde, observa-se grande variação e distribuição assimétrica, com medianas bem inferiores às médias (Tabela 3).

**Tabela 3:** Estatísticas descritivas de variáveis sociodemográficas e relativas à oferta de serviços da atenção primária e secundária em saúde bucal nas regiões de saúde do Brasil (N = 450), em 2023.

Variável	Média	DP	Mínimo	Q1	Mediana	Q3	Máximo
Quantidade de CEO	2,70	3,49	0	1	2	3	36
Quantidade de cadeiras odontológicas em CEO	13,96	23,99	0	3	7	15	206
Quantidade de cirurgiões-dentistas em CEO	32,08	53,11	0	7	17	36	607
População (habitantes)	451.291	857.163	25.660	148.082	252.968	422.692	11.451.999
Densidade demográfica (habitantes/km^2^)	129,09	490,60	0,55	13,88	35,12	71,45	7.529,26
Densidade eSB (eSB/100km^2^)	1,90	4,20	0,01	0,41	0,84	1,71	47,80
IVS	0,33	0,12	0,15	0,23	0,31	0,43	0,67

CEO: Centros de Especialidades Odontológicas; DP: desvio padrão; eSB: equipes de saúde bucal; IVS: Índice de Vulnerabilidade Social; Q: quartil.

Por fim, são apresentados os resultados do modelo *hurdle* binomial negativo, identificando fatores, no nível das regiões de saúde, associados à presença de CEO e à contagem de CEO, dada a sua presença (Tabela 4). Quando analisamos a parte de contagem, verificamos que IVS apresentou uma associação positiva estatisticamente significativa com o total de CEO por habitante (razão de taxas [RT] = 1,023; intervalo de 95% de confiança [IC95%]: 1,013-1,033). Este resultado indica que, entre as regiões que têm ao menos um CEO, cada centésimo de aumento no IVS está associado a um aumento de aproximadamente 2,3% na taxa de CEO relativa à população.

**Tabela 4:** Fatores associados à distribuição de Centros de Especialidades Odontológicas (CEO) nas regiões de saúde do Brasil, em 2023.

Parte contagem		
Variável	RT	IC95%
IVS	1,023	1,013-1,033
Densidade demográfica	0,983	0,971-0,996
Parte binária		
Variável	OR	IC95%
IVS	0,905	0,871-0,940
Região Norte	6,490	2,148-19,607
Região Nordeste	14,270	4,713-43,204
Região Centro-oeste	1,170	0,458-2,985
Região Sul	0,323	0,145-0,720
Densidade eSB	2,941	1,580-5,473

eSB: equipes de saúde bucal; IVS: Índice de Vulnerabilidade Social; IC95%: intervalo de 95% de confiança; OR: *odds ratio*; RT: razão de taxas estimada a partir do número de CEO por habitantes da região de saúde.

Por outro lado, a densidade demográfica mostrou uma associação negativa significativa (RT = 0,983; IC95%: 0,971-0,996), indicando que o aumento de uma centena de habitantes por km^2^ corresponde, em média, a uma redução de aproximadamente 1,7% no número de CEO por habitante. Quando analisamos a parte binária do modelo, que examina os fatores relacionados à presença (*vs.* ausência) de CEO, observa-se que um aumento de um centésimo no IVS está associado a uma redução de 9,5% nas chances de uma região de saúde dispor de CEO (*odds ratio* [OR] = 0,905; IC95%: 0,871-0,940).

Observam-se diferenças marcantes entre as macrorregiões brasileiras. Em comparação com a Região Sudeste (categoria de referência), as regiões de saúde no Nordeste e no Norte do país apresentaram chances significativamente maiores de dispor de CEO, com OR de 14,270 (IC95%: 4,713-43,204) e 6,490 (IC95%: 2,148-19,607), respectivamente, em condições semelhantes de vulnerabilidade social e densidade de eSB. A Região Centro-oeste não apresentou diferença estatisticamente significativa em relação ao Sudeste (OR = 1,170; IC95%: 0,458-2,985), enquanto a Região Sul mostrou chances significativamente menores (OR = 0,323; IC95%: 0,145-0,720), do que o Sudeste, de suas regiões de saúde disporem de CEO, sendo o IVS e a densidade de eSB semelhantes.

A densidade de eSB apresentou uma associação positiva e estatisticamente significativa (OR = 2,941; IC95%: 1,580-5,473) com o desfecho sugerindo que um aumento de 1 eSB por 100km^2^ está associado a um aumento de quase três vezes nas chances de a região de saúde possuir ao menos um CEO.

## Discussão

Os resultados revelam que a atenção secundária em saúde bucal vem rompendo com os padrões históricos de concentração de recursos nos centros mais desenvolvidos. Essa política redistributiva é observada na Região Nordeste, que dispõe de CEO em 93,23% das suas regiões de saúde e melhores indicadores de disponibilidade comparada ao Sudeste ^20^ ^,^ ^25^, o que vai de encontro a décadas de desenvolvimento socioeconômico desigual no Brasil. Os estados da Paraíba e do Rio Grande do Norte destacaram-se por apresentarem melhores indicadores regionais e pela presença da atenção secundária em saúde bucal em todas as suas regiões de saúde, demonstrando que é possível alcançar bons indicadores na oferta de serviços da atenção secundária em saúde bucal, mesmo em contextos de menor desenvolvimento econômico ^14^.

O Brasil, devido a sua grande extensão territorial e aos marcantes contrastes demográficos e socioeconômicos, apresenta desafios para a distribuição equitativa de serviços de saúde bucal ^4^ ^,^ ^25^. A análise da distribuição dos CEO revela como questões demográficas e socioeconômicas, assim como a estruturação dos serviços de saúde bucal na APS, impactam a oferta desses serviços ^20^ ^,^ ^25^. Contrastes são evidenciados, por exemplo, em áreas de alta e baixa concentração populacional, criando cenários completamente distintos para a organização dos serviços de saúde bucal. A Região Norte, com a menor densidade demográfica (4,51 habitantes/km^2^) e a maior extensão territorial, enfrenta o duplo desafio da baixa disponibilidade de CEO (0,44 CEO/100 mil habitantes) e das enormes distâncias geográficas. Este contexto exige estratégias diferenciadas que considerem não apenas a quantidade, mas também a localização estratégica dos CEO para maximizar o acesso populacional ^43^.

Outro desafio diz respeito à divisão heterogênea das UF em regiões de saúde; vale considerar o exemplo de dois estados da Região Centro-oeste, Mato Grosso do Sul e Goiás, com indicadores de oferta de CEO em relação à população similares. O Estado de Mato Grosso do Sul, apesar de dispor de CEO em todas as suas regiões de saúde, apresenta um número reduzido de regiões em sua vasta extensão de 357.103km^2^, o que implica grandes distâncias para o acesso aos serviços. Constitui um cenário menos favorável do que o apresentado pelo Estado de Goiás, que, apesar de não ter todas as suas regiões de saúde cobertas por CEO e de ter uma população 2,5 vezes superior, apresenta seu território dividido em um número maior de regiões de saúde. A divisão adequada em regiões de saúde e o planejamento territorial são decisivos para efetivar o acesso e superar desafios geográficos em estados extensos e em que a simples disponibilidade numérica de CEO, cadeiras odontológicas e cirurgiões-dentistas não garante acesso efetivo, sendo necessário considerar a acessibilidade geográfica na organização dos serviços de saúde ^30^.

A abordagem metodológica foi estruturada em consonância com as diretrizes de regionalização e hierarquização do SUS, visto que os CEO são serviços estratégicos para a estruturação da atenção secundária em saúde e que estes serviços deveriam estar disponíveis, minimamente, para atender à população de uma região de saúde. O modelo *hurdle* binomial negativo representa um avanço metodológico significativo no estudo de políticas de saúde bucal, incorporando uma sofisticação analítica apropriada à complexidade dos dados. Este modelo é particularmente adequado para lidar com o excesso de zeros (17,6% das regiões sem CEO) e permite a separação conceitual fundamental entre os dois processos distintos que governam a distribuição de CEO: os fatores que determinam se uma região terá pelo menos um CEO *versus* os fatores que influenciam quantos CEO existirão nas regiões que já têm o serviço.

O modelo de regressão múltipla em dois estágios revelou o padrão contraditório do IVS na implementação dos CEO, que seria impossível de detectar com modelos estatísticos tradicionais, pois permite que diferentes variáveis atuem de forma distinta em cada processo. As regiões de saúde com maior vulnerabilidade social apresentaram menores chances de ter CEO ^9^, no entanto, entre aquelas que apresentam o serviço implantado, observa-se que a vulnerabilidade social está associada a uma maior oferta de CEO. Este resultado alinha-se à “hipótese de equidade inversa” ^44^, segundo a qual a implementação de novas intervenções ou serviços tende, inicialmente, a ser disponibilizada aos segmentos mais favorecidos da população, gerando um aumento inicial das desigualdades (*top inequality*). Posteriormente, com o avanço da cobertura ou a criação de políticas para atender aos mais vulneráveis, ocorre a redução das desigualdades (*bottom inequality*) ^44^. Neste estudo, ainda que o recorte transversal não identifique um comportamento temporal, a menor implantação de CEO em regiões de alta vulnerabilidade sugere a existência de barreiras iniciais relacionadas à capacidade de gestão municipal e à infraestrutura ^25^. Entretanto, ultrapassada essa barreira, parece haver um esforço para aumentar o número de CEO para atender a uma população em situação de vulnerabilidade.

Apesar de a densidade demográfica ser um dos aspectos populacionais importantes para ajustar investimentos e a oferta de serviços no planejamento regional ^27^, e ser um dos requisitos para a implantação de CEO ^6^, observou-se um efeito negativo da densidade demográfica na quantidade de CEO (RT = 0,983 para cada 100 habitantes/km^2^), revelando que o aumento da densidade demográfica está associado a uma redução no número de CEO ^25^. Possivelmente, a acessibilidade geográfica se coloca como um elemento relevante no equacionamento ^4^. Além disso, certamente pesam outros fatores além do critério populacional, para a definição de uma maior oferta do serviço em uma região de saúde, como a capacidade administrativa, a presença de recursos físicos, humanos e financeiros, a cobertura de eSB e o quadro epidemiológico ^9^.

Este estudo indica que cada eSB adicional por 100km^2^ quase triplica as chances de uma região de saúde ter pelo menos um CEO, ou seja, a consolidação das eSB precede e/ou facilita a implantação de CEO, garantindo que exista uma base sólida de atenção primária em saúde bucal ^8^ ^,^ ^45^ para referenciar pacientes e dar suporte ao funcionamento de CEO, contribuindo para o princípio da integralidade no SUS. Esta integração entre CEO e eSB é importante para o fortalecimento das redes de atenção em saúde bucal ^28^.

Foram identificadas diferenças marcantes entre as macrorregiões do país no que concerne às chances de uma região de saúde dispor de CEO, com destaque para as maiores chances de presença de CEO no Nordeste e no Norte, e menores no Sul, em comparação ao Sudeste, considerando níveis de vulnerabilidade social e densidade de eSB semelhantes. Em alguma medida, esses achados podem sugerir a priorização da expansão de CEO nessas regiões nos últimos anos ^20^, bem como a implementação de mecanismos compensatórios para mitigar as inequidades em regiões mais desprovidas de CEO ^25^. Entretanto, não se pode ignorar, especificamente na Região Norte, a existência de menos regiões de saúde, com imensos territórios e desafios de acessibilidade relevantes. Por outro lado, a Região Sul surpreende com menor distribuição de CEO, em particular no Estado do Rio Grande do Sul ^23^, onde apenas 56,67% das regiões de saúde dispõem de um serviço, bem abaixo da média nacional. Embora se possa relativizar o fato de haver maior acessibilidade ao deslocamento na macrorregião, parece relevante um olhar sobre a necessidade de maior atenção à conformação das redes de atenção em saúde bucal, considerando, inclusive, a possibilidade de outros serviços de saúde oferecerem atenção especializada em saúde bucal ^21^.

Este estudo apresenta uma análise nacional da distribuição de CEO e oferece uma perspectiva alinhada à lógica organizacional do SUS, privilegiando a distribuição de CEO entre as regiões de saúde do Brasil e fornecendo subsídios para pensar sobre a importância da integração desses serviços ao planejamento regional. Espera-se, assim, contribuir para situar a regionalização da atenção à saúde bucal no contexto mais amplo da regionalização do SUS, com vistas à garantia de acesso equitativo aos serviços de atenção secundária em saúde bucal.

O estudo fornece contribuições relevantes para o entendimento da distribuição de CEO, cadeiras odontológicas e cirurgiões-dentistas da atenção secundária em saúde bucal, mas apresenta limitações importantes inerentes à análise exclusiva da dimensão estrutural dos serviços, pois não capta outros aspectos importantes dos serviços de saúde, como o acesso e a utilização, a qualidade do cuidado, a integração entre níveis de atenção, aspectos de gestão e os resultados. Além disso, por depender da iniciativa do gestor municipal ou estadual, não é possível mensurar se o número de CEO está alinhado aos objetivos da PNSB. A utilização de dados secundários, embora permita realizar uma análise nacional abrangente, pode subestimar ou superestimar a realidade assistencial devido a inconsistências no cadastramento. O IVS apresenta defasagem temporal, e transformações socioeconômicas podem ter alterado o perfil de vulnerabilidade das regiões de saúde. Outra limitação refere-se à agregação por regiões de saúde, que pode minimizar as diferenças entre os municípios de uma mesma região de saúde, visto que estes podem apresentar padrões distintos de vulnerabilidade.

Apesar dos avanços, a existência de 17,6% das regiões de saúde sem qualquer CEO indica persistência de vazios assistenciais significativos. Paralelamente, a literatura documenta a subutilização da capacidade instalada de CEO ^18^ ^,^ ^19^, o que sugere deficiências na articulação entre a atenção primária e secundária, falhas nos sistemas de referência e contrarreferência e possíveis inadequações na gestão dos serviços. A resolução desses problemas requer não apenas a expansão quantitativa ^21^, mas, sobretudo, o planejamento e a otimização dos fluxos assistenciais. Os achados reforçam a necessidade de abordagens regionalizadas no planejamento da saúde bucal ^24^ ^,^ ^25^ ^,^ ^26^ ^,^ ^27^. A heterogeneidade identificada exige estratégias diferenciadas, e a Região Nordeste pode servir como um modelo de implementação bem-sucedido, pelo menos do ponto de vista da capacidade de oferta de serviços ^8^ ^,^ ^20^ ^,^ ^28^ ^,^ ^45^.

## Considerações finais

Os resultados evidenciam que a distribuição de CEO vem alcançando regiões mais vulneráveis do país e rompendo com padrões históricos de concentração de recursos. Embora se observe que a implementação de CEO ocorra em regiões menos vulneráveis, estão sendo desenvolvidas estratégias compensatórias para que esses serviços estejam disponíveis à população de regiões mais vulneráveis (hipótese da equidade inversa). Contudo, persistem desafios significativos relacionados à dimensão territorial brasileira, a vazios assistenciais e à necessidade de otimizar a utilização dos recursos instalados. Ainda que seja necessário pensar a atenção secundária em saúde bucal em um contexto regionalizado, a desigual distribuição de regiões de saúde entre as UF e as macrorregiões do país, a grande variação de suas áreas territoriais e os problemas de mobilidade nos territórios podem dificultar o acesso aos CEO.

A utilização dos CEO não foi objeto deste trabalho, mas constitui um campo importante para pesquisas futuras, pois a efetivação do acesso universal e equitativo à atenção secundária em saúde bucal requer não apenas expansão quantitativa. É necessário entender como os CEO estão sendo utilizados pela população para desenvolver estratégias que fortaleçam a rede de atenção em saúde bucal. Também são necessários estudos sobre os fatores relacionados à falta de interesse por parte dos gestores municipais e estaduais na implantação de CEO.

Ainda que a atenção secundária em saúde bucal ocorra em um cenário de distribuição desigual de recursos, os achados indicam redução das inequidades.

## Data Availability

Os bancos de dados utilizados no estudo, incluindo as informações sobre a extração e análises estão disponíveis em repositório: https://data.mendeley.com/datasets/fb22pnxkyr/2.
